# American Exceptionalism: Population Trends and Flight Initiation Distances in Birds from Three Continents

**DOI:** 10.1371/journal.pone.0107883

**Published:** 2014-09-16

**Authors:** Anders Pape Møller, Diogo S. M. Samia, Mike A. Weston, Patrick-Jean Guay, Daniel T. Blumstein

**Affiliations:** 1 Laboratoire d'Ecologie, Systématique et Evolution, Centre National de la Recherche Scientifique Unité Mixte de Recherche 8079, Université Paris-Sud, Orsay, France; 2 Ecology and Evolution, Laboratory of Theoretical Ecology and Synthesis, Federal University of Goiás, Goiânia, Brazil; 3 Centre for Integrative Ecology, School of Life and Environmental Sciences, Faculty of Science, Engineering and the Built Environment, Deakin University, Melbourne, Victoria, Australia; 4 College of Engineering and Science, and Institute for Sustainability and Innovation, Victoria University, Footscray Park Campus, Melbourne, Victoria, Australia; 5 Department of Ecology & Evolutionary Biology, University of California Los Angeles, Los Angeles, California, United States of America; Institute of Agronomy, University of Lisbon, Portugal

## Abstract

**Background:**

All organisms may be affected by humans' increasing impact on Earth, but there are many potential drivers of population trends and the relative importance of each remains largely unknown. The causes of spatial patterns in population trends and their relationship with animal responses to human proximity are even less known.

**Methodology/Principal Finding:**

We investigated the relationship between population trends of 193 species of bird in North America, Australia and Europe and flight initiation distance (FID); the distance at which birds take flight when approached by a human. While there is an expected negative relationship between population trend and FID in Australia and Europe, we found the inverse relationship for North American birds; thus FID cannot be used as a universal predictor of vulnerability of birds. However, the analysis of the joint explanatory ability of multiple drivers (farmland breeding habitat, pole-most breeding latitude, migratory habit, FID) effects on population status replicated previously reported strong effects of farmland breeding habitat (an effect apparently driven mostly by European birds), as well as strong effects of FID, body size, migratory habit and continent. Farmland birds are generally declining.

**Conclusions/Significance:**

Flight initiation distance is related to population trends in a way that differs among continents opening new research possibilities concerning the causes of geographic differences in patterns of anti-predator behavior.

## Introduction

Many species are currently declining or going extinct in what has been called the sixth mass extinction [1]. The main culprits for this dire situation are the multiple deleterious processes caused or promoted by humans that include habitat modification and fragmentation, over-exploitation of natural resources, pollution, introduced species, and climate change [2].

Human activity and infrastructure, and the various stimuli and chemicals we produce, is omnipresent, affecting even the most remote parts of the planet. Given the history of human exploitation of, or interference with a large fraction of living organisms, it is no surprise that human disturbance (disruption of ‘normal’ states) constitutes a major potential impact. Among most animals, especially vertebrates, but also invertebrates (reviews in [3]), such disruption may occur physiologically in the form of changes to heart beat or core temperature, and/or behaviorally, in the form of escape [4]. Most animals take flight when approached by humans, and the distance at which this takes place in response to humans or other predators is termed the flight initiation distance (FID). This simple behavioral measure of susceptibility to human proximity and approach reflects an animal's compromise between benefitting from remaining in-situ in terms of time spent foraging and conservation of energy, and the cost of risk of predation and death (reviewed in [3]). Disturbance may result in fitness costs with consequences in terms of reduced rates of reproduction and survival, and these may eventually manifest themselves at the population level as negative population growth [5]. Accordingly, population trends of common breeding birds in Europe are negatively related to relative FID, even when controlling statistically for potentially confounding drivers of population trends such as habitat loss, cognitive ability and climate change [6].

Many national and international organizations are monitoring the population trends of organisms as diverse as birds, mammals, butterflies and bumblebees. In particular, birds have been monitored since the 1960's in many countries in Europe and North America, and continent-wide monitoring takes place in North America and Europe. Elsewhere, such as Australia, atlases permit some assessment of temporal trends (Dunn and Weston 2008). Of great concern are widespread reports of species declines, and understanding the drivers of change in bird populations is key to managing these. Recently, Reif [7] reviewed drivers of long-term population trends in Europe concluding that human impacts were the main factors. Currently, studies of comparisons of drivers of change in among countries or continents are rare. Møller et al. [8] showed negative effects of climate change on population trends in birds across Europe. Pocock [9] demonstrated negative effects of agriculture in both North America and Europe. Møller [6] found a correlation between population trends and FID of different species of birds in Europe, while Thaxter et al. [10] did not find such a relationship for Danish FID and English population trends. Bennett and Owens [11] showed that larger species are more often threatened. A particularly revealing study by Reif et al. [12] showed that species with a relative large brain size differed in population trends across the ‘iron curtain’ between Western and Eastern Europe during the post World War II decades. There were negligible effects of relative brain size on population trends in Western Germany, slightly positive effects in Eastern Germany and strongly positive effects in the Czech Republic. These intriguing and varying patterns, and the need to optimize conservation priorities, means there are good reasons to investigate patterns of population trends at different spatial scales in an attempt to elucidate important correlates. This diversity of factors associated with population trends, and in particularly the heterogeneity in effects among studies, is intriguing begging the question whether these patterns reflect random noise, robust drivers of population trends, or heterogeneous drivers that reveal biologically meaningful effects.

Here we test if population trends across three continents differing in their histories of human impact can be explained by susceptibility to human disturbance. We focus on the disruption of behavior induced by the presence of an approaching person. The objectives of this study were to: (1) quantify the magnitude of the effect of response to human disturbance as reflected by FID on population trends; (2) test for differences in relationships between population trends and FID among three continents (North America, Australia and Europe) and that such differences may be due to differences in FIDs among species and continents; and (3) test for the joint effect of multiple drivers on population trends including farmland habitat, body mass, migration distance and pole-most breeding latitude. To this end we used information on FID, population trends and potential drivers of these trends relying on our studies of FID in 193 species of birds from three continents.

## Methods

### Flight Initiation Distance (FID)

We recorded FID for a total of 238 species, which was later reduced to 193 because of missing values for some variables, by using a standard procedure developed by Blumstein [13]. There were very few species that occurred in more than a single continent. FID in Europe were recorded in Norway, Denmark, France and Spain. In brief, we walked at ordinary walking speed directly towards a bird recording the distance from the bird when we started walking, the distance at which the birds initiated escape, and the bird's height in the vegetation. This information was used to estimate the FID. In order to account for the height in which individuals were perched, FID was calculated as the Euclidian distance between the approaching human and the focal bird (which equals the square-root of the sum of the squared flight distance and the squared height in the vegetation). We also recorded starting distance (the distance between the observer and the individual bird being observed when the approach to the bird was initiation) between an observer and focal individual birds, although we did not report starting distances here for simplicity. We did not consider starting distance here because it may arise as an artefact of choice of study method. Observers wore neutral colored clothes and behaved as normal pedestrians. FIDs were recorded in a representative range of habitats by searching systematically for birds in all available habitats. FID was measured by a number of trained observers and therefore data were pooled for analysis [14]. The FID estimates were initially reported in Blumstein [13], Møller [6], and Weston et al. [15].

No specific permissions were required for these locations/activities. The field studies did not involve endangered or protected species and Blumstein [13], Møller [6], and Weston et al. [15] provide further details. The collection of FID data only required behavioral observations that did not involve capture, collection or sacrifice of any specimens.

### Population Trends

We obtained population trends for the years 1980–2012 for breeding birds relying on the European Bird Census Council (http://www.ebcc.info/index.php?ID=509). We used the US Audubon Society's Christmas Bird Count data to calculate trends for North American species for the years 1990–2012. We regressed year and number of sites reporting, on N birds counted, and interpreted the coefficient estimates of year as the population trend. The vast majority of FID data were collected in California; California and continental trends were positively correlated (Pearson *r* = 0.45), so we used the California trend for 2000–2010 for the analyses. Assessing population trends in Australia is difficult, although two continental scale Atlases exist [16]. A formal comparison between the reporting rates (i.e. an index of abundance) of these two Atlases (1977–1981 vs. 1998–2002) describes the best available trends at the continental scale for many species, and included consideration of regional variation in trends [16], [17]. For this study, we selected only those species with regionally consistent trends (i.e. non-significant regional variation) because FIDs came from various regions, and many FIDs could not be reliably geo-referenced to region. The vast majority of Australian FIDs were collected in southeastern and eastern Australia. The trends from each continent were standardized to a mean of zero to allow merging of the data. We did not standardize the variance in trends to allow for trends to vary among continents.

### Ecological Variables

We selected five variables, which have been implicated in population change among birds, to include in our models. These were:

1. **Farmland breeding habitat.** All species were scored as breeding in farmland habitat or other habitats using the habitats listed by the European Bird Census Council (http://www.ebcc.info/index.php?ID=509). For North American birds we used Small [18], and for Australian birds we used Pizzey and Knight [19] supplemented with the Handbook of Australian, New Zealand and Antarctic Birds series [20].
2. **Pole-most latitudinal range.** We recorded the northernmost breeding latitude for Europe and North America and the southernmost breeding latitude for Australia relying on standard handbooks or atlases [17], [20]–[22].
3. **Migration.** We scored all species as migrants or residents relying on standard handbooks or field guides [21]–[23], listing species as residents if the range occupied by the bulk of the population overlapped during breeding and during winter.
4. **Body mass.** We used information on body mass of adults relying on standard handbooks averaging the body mass of males and females if body masses were reported separately for the two sexes [20]–[22]. All data for different species in different continents are reported in File S1.

### Comparative Analyses

Closely related species are more likely to have similar phenotypes because of common ancestry, which makes data points statistically dependent [24]. We fitted a series of phylogenetic generalized least-squared models (PGLS; [25]) to evaluate the impact of phylogenetic relationships on the relationship between FID and population trends. We fitted four sets of PGLS that modeled different evolutionary scenarios to test the robustness of our findings: (1) assuming that trait evolved under a Brownian motion model of evolution (i.e. assuming a Pagel's λ = 1) [26]; (2) transforming the branch length of the phylogenetic tree (optimizing the Pagel's λ by maximum likelihood) so that it reflected the strength of the phylogenetic signal of the trait [26]; (3) assuming that the trait evolved under an Ornstein-Uhlenbeck (OU) model of evolution (i.e. assuming α = 1) [27]; and (4) transforming the branch length of the phylogenetic tree (optimizing the α parameter by maximum likelihood) assuming an OU model of evolution so that it reflects the strength of the phylogenetic signal in the trait [27]. These different evolutionary scenarios were simulated by transforming the variance/covariance matrix of the data given the specific phylogeny [25] using the R package “ape” [28] and then including the correlation structure of the model using the “gls” function of the R package “nlme” [29]. In all cases (following our Ordinary Least Squares (OLS) methods), all PGLS models were weighted by sample size to account for differences in sample size among species [30], [31]. To do so, we used the inverse of the sample size as a proxy of variance to be used in the variation function structure (argument “weights” of the “gls” function; [28]).

We used the most recent avian super-tree [32], http://birdtree.org/) to reconstruct the evolutionary history of the species included in our data set. We used two phylogenetic trees in our analyses to test if our conclusions were sensitive to the choice of phylogeny: the Ericson backbone and the Hackett backbone phylogenies (File S2, S3).

In addition to the main effects, we tested for significant interactions between continent and the other five predictor variables, while maintaining the main effects aiming to test if variables predict population trends in a different way depending on the continent where the bird species live. We also tested for the interaction between FID and body mass because FID may vary with body mass. Our candidate models comprise all possible combination among these terms. A constant term (intercept) was included in all models. Our candidate models respected “marginality constraints” so that models containing interactions were not included without their respective main effects. In total, the five sets of analyses (i.e., OLS plus four PGLS models) produced 391 candidate models each.

We used an information theoretic approach based on Akaike's criteria corrected for small sample size (AICc) to evaluate the set of candidate models (OLS models and four scenarios of the PGLS models) [32]. AICc is a measure of distance of putative model from full reality [37]. The candidate models are ranked by their AICc values. The best model was that with the lowest AICc value. However, models with ΔAICc<2 are considered equally good as models with the lowest AICc. Given the low relative likelihood of our best models as indicated by their Akaike weights (*w*_i_<0.9; Table 1), we employed a multi-model inference approach [33]. We computed averaged estimates of the predictors across the 391 candidate models by weighting their estimates by the *w*_i_ of the models in which they were included [33]. We also calculated the relative importance of each predictor by summing the *w*_i_ over all models in which they were included [33]. Importance ranges from 0 to 1; larger values indicate greater importance of a predictor to explain the population trends of birds.

**Table 1:** Predictors and the three best Ordinary Least Squares models (i.e. with ΔAICc<2) explaining the variation in the population trends of birds.

Factor	1	2	3	*b* (SE)	Importance
Intercept	•	•	•	−0.886 (1.076)	1.000
Continent	•	•	•	+	0.999
Farmland	•	•	•	−0.610 (0.364)	0.997
FID	•	•	•	2.087 (1.254)	0.995
Continent: FID	•	•	•	+	0.984
Body mass	•	•	•	−1.701 (0.826)	0.961
Continent: Body mass	•	•	•	+	0.881
FID: Body mass	•	•	•	+	0.879
Migration		•	•	−0.039 (0.196)	0.474
Latitude				−0.002 (0.008)	0.342
Continent: Migration			•	+	0.207
Continent: Latitude				+	0.121
Continent: Farmland				+	0.090
k	8	9	10		
AICc	596.2	597.5	597.9		
ΔAICc	0.00	1.30	1.74		
*w*_i_	0.261	0.136	0.109		
R^2^	0.27	0.27	0.29		

Variables included in the models are indicated by a filled circle (•). The number of parameters (k), AICc, ΔAICc, Akaike weight (*w*_i_), and coefficient of determination (R^2^) are shown below each model. Model averaged estimate (*b*) among the 391 candidate models and the relative importance are shown for each predictor. “+” symbol indicates factors with more than one level. See Methods for further details.

## Results

Our data set consisted of 193 species with full data on all variables out of an initial 238 species. Mean FID did not differ significantly among continents (Welch ANOVA for unequal variances: *F* = 1.29, d.f. = 2, 00.77, *P* = 0.28), nor were the variances significantly different (Levene's test: *F* = 2.00, d.f. = 2, 190, *P* = 0.14). Mean body mass differed significantly among continents with the mean being the smallest in Europe followed by North America and Australia (Welch ANOVA for unequal variances: *F* = 7.79, d.f. = 2, 80.72, *P* = 0.0008), but the variances did not differ significantly (Levene's test: *F* = 1.62, d.f. = 2, 190, *P* = 0.20).

Our model selection showed that the OLS model was more parsimonious than the four sets of PGLS models (regardless of the phylogeny used; File S4, S5, S6, S7, S8, S9, S10, S11, S12). The ΔAICc between the best OLS models (Table 1) and the best PGLS models (i.e. that with optimized λ) were at least 13.89. The superiority of the OLS models is given by the absence of phylogenetic signal both in response variable and, even more importantly, in residuals (maximum-likelihoods: λ = 0 and α>128527). Therefore, we used the OLS models to make our inferences.

**Figure 1:**
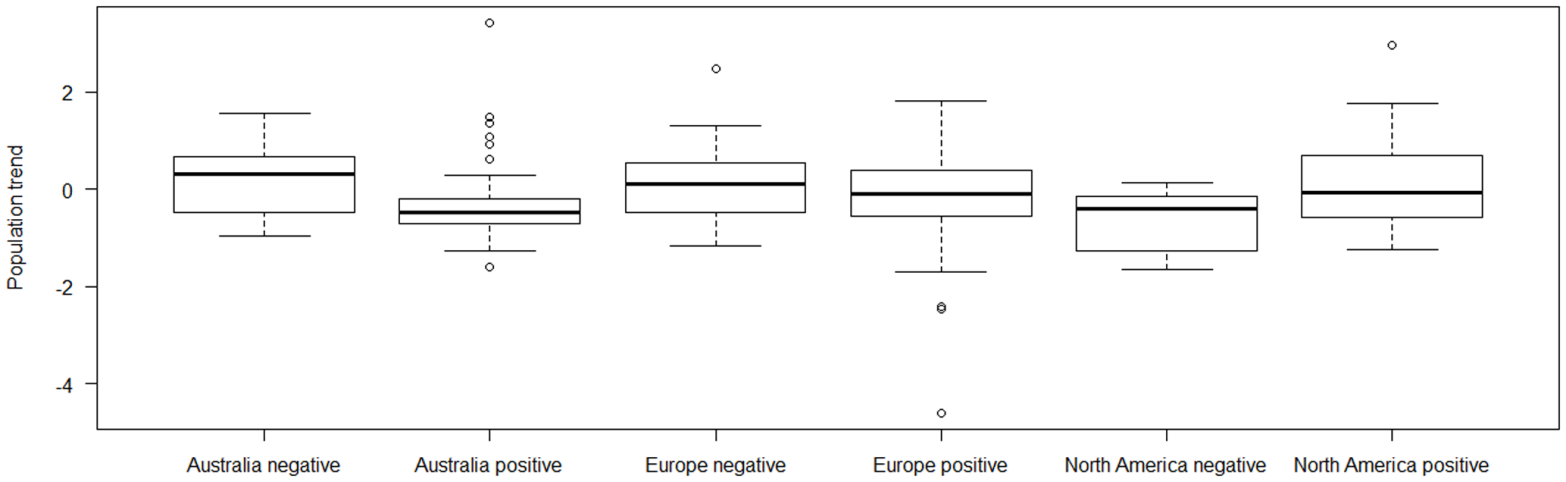
Box plots of population trends for species with negative and positive relative flight initiation distances in Australia, Europe and North America. The box plots show median, quartiles, 5- and 95-percentiles and extreme values. Relative flight initiation distances were residuals from a regression of log_10_-transformed FID on log_10_-transformed body mass, and species were split into two similarly sized categories with negative and positive residuals, respectively, in order to illustrate the difference in population trends between species with relatively short and relatively long FIDs for their body size. We emphasize that this was only done for illustrative purposes.

**Figure 2:**
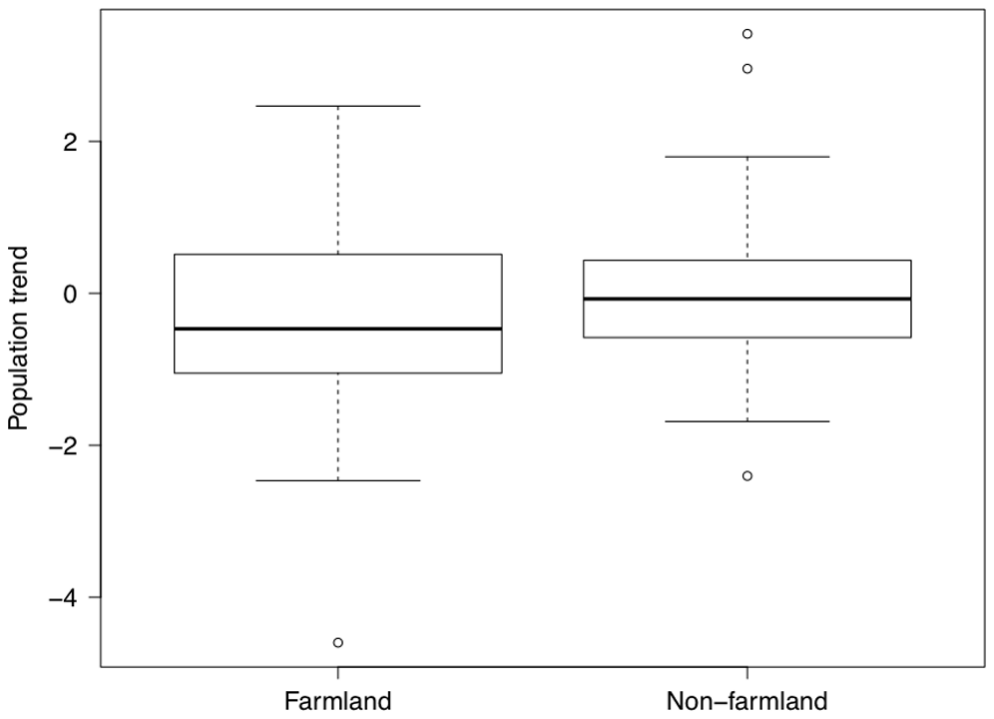
Box plots of population trends for farmland and non-farmland species. The box plot shows median, quartiles, 5- and 95-percentiles and extreme values. NB: Only two Australian and two North American species were classified as farmland species.

Based on the ΔAICc criteria, we selected three models as the most parsimonious to explain population trends of birds. These models explained between 27% and 29% of the variance of the data. Population trend was correlated with FID in the models that also included effects of continent, farmland breeding habitat, body mass and migration (Table 1). Indeed, continent, farmland breeding habitat, FID, body mass, the interaction between continent and FID, the interaction between continent and body mass, and between FID and body mass were present in all of the three best models, all with importance over 87%. Bird species from Australia and Europe had negative population trends when their FID was long for a given body size, and hence they had a positive residual FID, while the opposite was the case for bird species from North America (Fig. 1). Population trends for farmland birds were generally negative, while that was not the case for species with other habitats (Fig. 2). Despite an overall relatively low weight (47%), we found significant effects of migration in two of the best models (Table 1). All the models are reported in File S4, S5, S6, S7, S8, S9, S10, S11, S12.

## Discussion

The main findings of this intercontinental study of population trends of birds were that species with declining populations were mainly farmland migrants (though there were few farmland species in our final, comprehensive, data set that lived in Australia and North America). Moreover, we found a significant interaction between continent and flight initiation distances. There was a large effect of FID and the direction of the effect varied significantly among continents as shown by the interaction. While bird populations are declining with increasing FID in Australia and Europe, the opposite was the case in North America. There were also significant interactions between body mass and continent and between body mass and FID. These correlations are open to interpretation, and they potentially have important implications.

The relationships between population trends and FID differed among continents with North America being an exception for the pattern found in Australia and Europe, hence American exceptionalism. The continental variation was somewhat unexpected and we do not have any a posteriori explanation for it. Pocock [9] analyzed data of population trends of birds from eight regions in two continents, finding that population trend relationships from one region are poor predictors of population trend relationships in another. Our findings on heterogeneity in the relationship between FID and population trends fit well into this scenario, cautioning against using spatially unreplicated findings as a basis for decision-making such as conservation policy. It is important to note that effect sizes in biological sciences generally are small to intermediate only explaining 5–10% of the variance [34]. Hence there is little prospect for making truly predictive models with such low coefficients of determination.

Many drivers of population trends of birds have been proposed and documented [7], and we included these as potentially confounding variables in our analyses. These confounding variables range from migration [7], [35]–[37], brain mass [8], [12], [38], thermal maximum and number of broods [39], [40] to body mass [11]. We found evidence of migration, farmland breeding habitat driven by European birds. We could not include brain mass and body mass in the same models because of collinearity, and hence we excluded brain mass from subsequent models because this variable had the largest number of missing values. However, the main conclusion of significant continent-specific effect of FID on population trends remained even after controlling statistically for these potentially confounding variables with high importance among the candidate models.

While we have no explanation for the trend observed in North America, we can exclude a number of candidate explanations. All estimates of FID were made in similar and highly consistent ways. Indeed, studies of FID have shown consistency in FID among observers in different countries, among observers in the same locality, within observers among localities, and within observers among seasons and years [8], [41], [42]. We can also exclude the possibility that the differences among continents were due to differences in means or variances of FID and body mass among continents. The frequency distributions of FID and body mass were relatively similar with differences among continents reflecting small effect sizes. FID can vary with the degree of exposure to humans, but North American human population density fell within the bounds of the other two continents; 72.6 inhabitants km^−2^, Europe, over 31.7 inhabitants km^−2^ in the USA and 3.04 inhabitants km^−2^ in Australia, even though our FID data collection took place in parts of the continents with a higher than average population density. Each continent has its complement of aerial and terrestrial predators (flight is an anti-predator response [3]. Irrespective of the reason for these differences in the relationship between FID and population trends among continents, we can infer that FID as a behavioral measure of susceptibility to human disturbance has different meaning or different information content in different continents. Assuming that the FID-population trend relationship varies within a species as it does between species, in some continents or areas it may be a useful proxy for the risk of population change. Certainly, FID would be cheaper and easier to monitor than long-term population trends.

Bird species breeding in farmland displayed the steepest declines as agriculture has become ever more industrialized and intensified and thereby disproportionately affects farmland specialists [7]–[8], [43]–[44]. Here we found evidence consistent with this general trend with ‘specialist’ farmland species having the strongest population declines, even when controlling statistically for other potentially confounding variables.

Climate change has affected the distribution of many species, and range margins have on average moved pole-wards [45]. However, we found little evidence of pole-most breeding latitude being related to population trends, consistent with other studies [7]. Cuervo and Møller [46] have recently shown that fluctuations in population size of breeding birds in Europe are the strongest at the margins of the breeding distribution, but are particularly variable at the southern-most range margins, where increasing temperatures may render environmental conditions for maintenance of viable populations the most difficult.

In conclusion, we have shown that population changes of birds are related to FID in a continent-specific manner with species with the longest FIDs having the steepest declines in Europe and Australia, while the opposite pattern was found in North America. In addition, there were independent effects of farmland breeding habitat, bird migration, body mass and other variables on population trends of birds. However, the main findings of continent-specific relationships of FID on population trends remained robust, as did the relationship for farmland breeding habitat and body mass.

## Supporting Information

File S1 **Full dataset used in the analyses.**
(XLSX)

File S2 **Phylogeny based on Hackett** [32] **.**
(PDF)

File S3 **Phylogeny based on Ericson** [32] **.**
(PDF)

File S4 **OU optimized models based on Hackett** [32] **.**
(XLSX)

File S5 **OU fixed models based on Hackett** [32] **.**
(XLSX)

File S6 **Lambda models based on Hackett** [32] **.**
(XLSX)

File S7 **BM models based on Hackett** [33] **.**
(XLSX)

File S8 **OU optimized models based on Ericson** [32] **.**
(XLSX)

File S9 **OU fixed models based on Ericson** [32] **.**
(XLSX)

File S10 **Lambda models based on Ericson** [32] **.**
(XLSX)

File S11 **BM models based on Ericson** [32] **.**
(XLSX)

File S12 **OLS models.**
(XLSX)
