# Supplementary figures and images for: American Exceptionalism: Population Trends and Flight Initiation Distances in Birds from Three Continents
Source: PLoS One. 2014 Sep 16;9(9):e107883. doi: 10.1371/journal.pone.0107883 (PMC4166455; doi:10.1371/journal.pone.0107883)

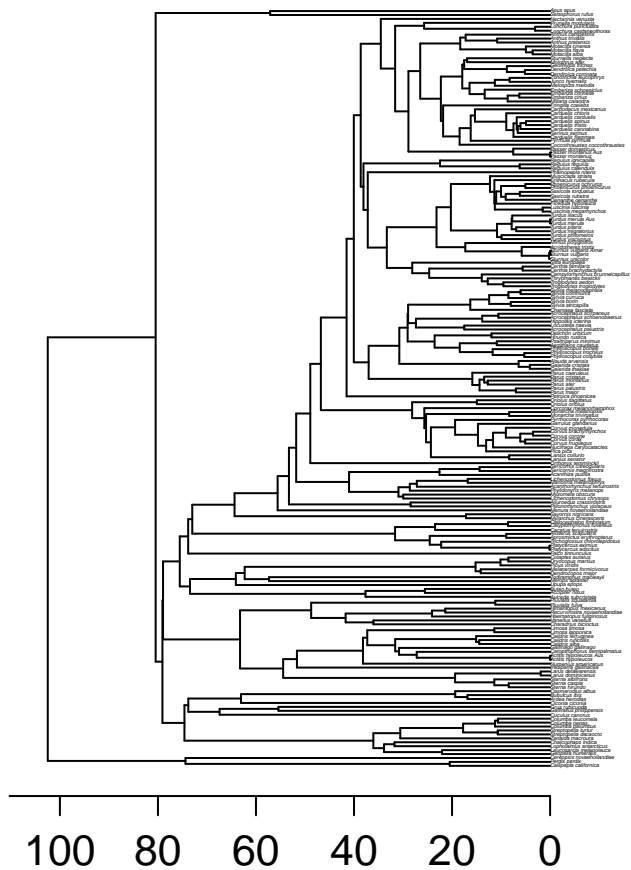

Supplement: File S2 — Phylogeny based on Hackett [32] . (PDF) [file pone.0107883.s002.pdf]

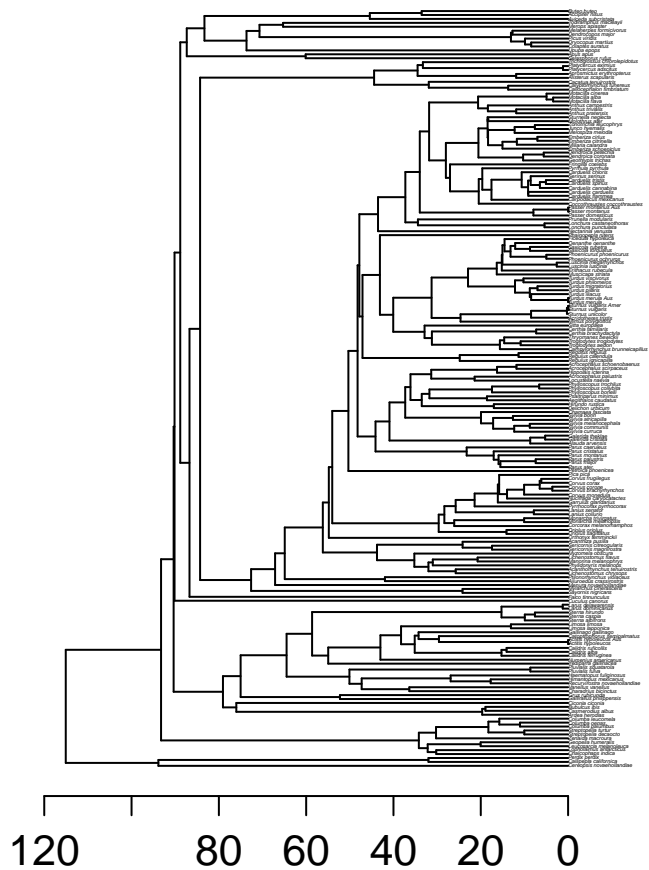

Supplement: File S3 — Phylogeny based on Ericson [32] . (PDF) [file pone.0107883.s003.pdf]
